# The Suppression of Columnar π-Stacking in 3-Adamantyl-1-phenyl-1,4-dihydrobenzo[*e*][1,2,4]triazin-4-yl

**DOI:** 10.3390/molecules21050636

**Published:** 2016-05-14

**Authors:** Christos P. Constantinides, Andrey A. Berezin, Georgia A. Zissimou, Maria Manoli, Gregory M. Leitus, Panayiotis A. Koutentis

**Affiliations:** 1 Department of Chemistry, University of Cyprus, P. O. Box 20537, Nicosia 1678, Cyprus; berezin.andrey@ucy.ac.cy (A.A.B.); georgia_zissimou@hotmail.com (G.A.Z.); manoli.maria@ucy.ac.cy (M.M.); 2 Department of Chemical Research Support, Weizmann Institute of Science, Rehovot 76100, Israel; gregory.leitus@weizmann.ac.il

**Keywords:** 1,2,4-benzotriazinyls, hydrazyls, adamantyl, paramagnet, organic radicals, crystal packing

## Abstract

3-Adamantyl-1-phenyl-1,4-dihydrobenzo[*e*][1,2,4]triazin-4-yl (**4**) crystallizes as chains of radicals where the spin bearing benzotriazinyl moieties are isolated from each other. Magnetic susceptibility studies in the 5–300 K temperature region indicate that radical **4** demonstrates typical paramagnetic behavior stemming from non-interacting *S* = ½ spins.

## 1. Introduction

**Chart 1:**
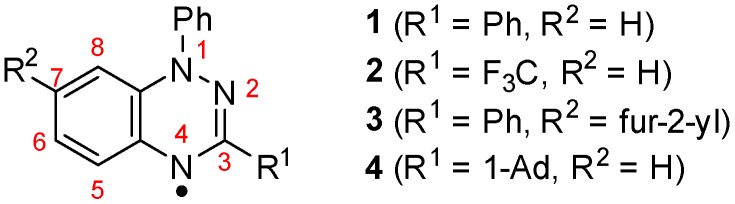
Structures of 1,2,4-benzotriazin-4-yls **1**–**4** and IUPAC numbering of the ring system.

1,4-Dihydrobenzo[*e*][1,2,4]triazin-4-yls a sub-class of the hydrazyl family of radicals has attracted considerable attention owing to their robust thermal, moisture and air stability. The first 1,2,4-benzotriazinyl radical was reported by Blatter in 1968 (Blatter radical **1**, Chart 1) [1], and, aside from electrochemical studies by Neugebauer [2,3,4,5], this stable radical remained relatively obscure. Nevertheless, the recent development of improved syntheses of 1,2,4-benzotriazinyls [6,7,8,9,10] have led to numerous new derivatives, some of which display a range of low dimensional magnetic properties [11,12,13,14,15,16,17,18,19,20,21,22], while others have been used as radical initiators in polymer chemistry [23,24,25], and as sensors of picric acid [18].

Solid-state σ or π dimerization is suppressed in the majority of these radicals, nevertheless, for two derivatives radicals within the π-stacked columns associate leading to dimers with singlet ground states: (a) 1-phenyl-3-trifluoromethyl-1,4-dihydrobenzo[*e*]-[1,2,4]triazin-4-yl (**2**) demonstrates an abrupt fully reversible spin transition at 58(2) K between a diamagnetic low temperature phase and a paramagnetic high temperature phase [16] and (b) 7-(fur-2-yl)-1,3-diphenyl-1,4-dihydrobenzo[*e*][1,2,4]triazin-4-yl (**3**) has a singlet ground state below 100 K and a thermally accessible triplet state [13].

**Figure 1:**
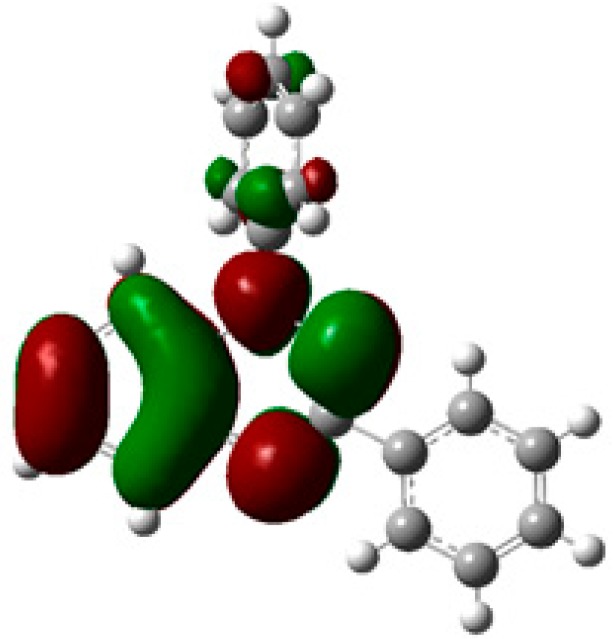
SOMO of Blatter radical **1** calculated at the UB3LYP/6-311+G(d,p).

The extensive delocalization of the singly occupied molecular orbital (SOMO) orbital density over the benzo-fused hydrazonyl and the N1-Ph (Figure 1), indicates that positive orbital overlap and π-stacking, can be achieved in a variety of different association modes, and a number of different solid-state packing motifs have been reported [11,12,13,14,15,16,17,18,19,20,21,22]. In the majority of crystals structures reported to date, 1,2,4-benzotriazinyls π stack to form 1-D columns. The propensity of 1,2,4-benzotriazinyls to form columns could potentially be overcome by strategic introduction of bulky substituents in the periphery. To test this hypothesis we prepared a 1,2,4-benzotriazinyl substituted with an adamantyl group at C-3. Herein, we report the crystal structure and magnetic properties of the 3-adamantyl-1-phenyl-1,4-dihydrobenzo[*e*][1,2,4]triazin-4-yl (**4**).

## 2. Results and Discussion

### 2.1. Synthesis, Electrochemical and EPR Data

**Scheme 1:**
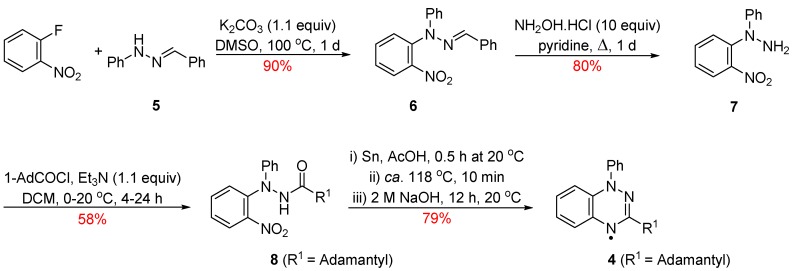
Synthesis of the 3-adamantyl-1,2,4-benzotriazinyl **4**.

Radical **4** was previously prepared via a multistep route (Scheme 1) starting from the readily available 1-benzylidene-2-phenylhydrazine (**5**) [10]. Reaction of the benzylidene **5** with 1-fluoro-2-nitrobenzene and K_2_CO_3_ in DMSO at *ca.* 100 °C for 1 day gave 2-benzylidene-1-(2-nitrophenyl)-1-phenylhydrazine (**6**) which upon treatment with excess NH_2_OH·HCl in pyridine and heating to *ca.* 80 °C for 1 day released 1-(2-nitrophenyl)-1-phenylhydrazine (**7**) in 80% yield. Acetylation and subsequent mild reduction of the nitro group followed by an acid-mediated cyclodehydration gave the fused triazine which upon alkali treatment afforded the desired adamantly-substituted radical **4**.

**Figure 2:**
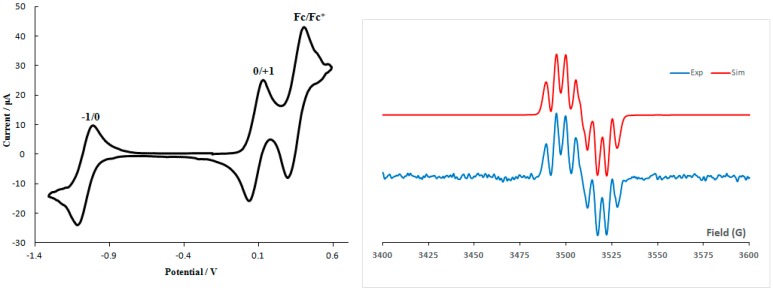
Cyclic voltammetry (**left**) and solution EPR spectrum (**right**) of radical **4**.

The redox behavior of radical **4** is typical of 1,2,4-benzotriazinyls exhibiting two fully reversible waves corresponding to the −1/0 and 0/+1 processes (Figure 2, left) [11,12,13,14,15,16,17,18,19,20,21,22]. Worthy of note is that the oxidation potential of *E*_1/2_^0/+1^ = 0.08 V *vs* Fc/Fc^+^ couple makes radical **4** the best 1,2,4-benzotriazinyl donor reported to date. The solution EPR spectrum of radical **4** (Figure 2, right) exhibits the typical 1,2,4-benzotriazinyl seven-line multiplets consistent with coupling of the unpaired electron with the three similar but slightly nonequivalent ^14^N nuclei. The hyperfine coupling constants (hfcc), determined by the simulation of the EPR spectrum, are *a*_N(1)_ = 7.51, *a*_N(2)_ = 4.94 and *a*_N(4)_ = 5.13 G with g = 2.0040.

### 2.2. X-ray Studies

**Figure 3:**
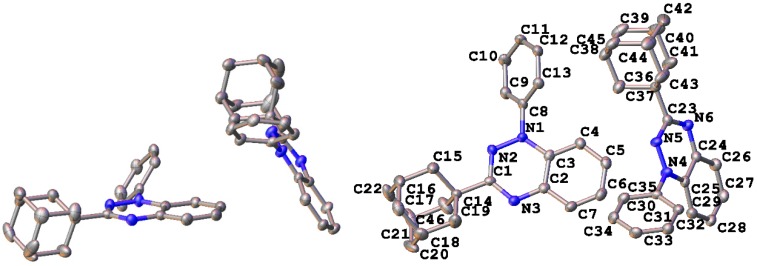
Asymmetric unit (**left**) and crystallographic atom numbering for 3-adamantyl-1,2,4-benzotriazinyl **4** (**right**). Hydrogen atoms omitted for clarity.

Suitable single crystals of radical **4** for X-ray diffraction studies were obtained by slow cooling of a concentrated *c*-hexane solution. Radical **4** adopted the orthorhombic space group *Pna*2_1_ with two molecules in the asymmetric unit. These two molecules are oriented in a dihedral angle of 56.9° with respect to each other (Figure 3). The intramolecular geometry of radical **4** is typical of other 1,2,4-benzotriazinyls [11,12,13,14,15,16,17,18,19,20,21,22].

**Figure 4:**
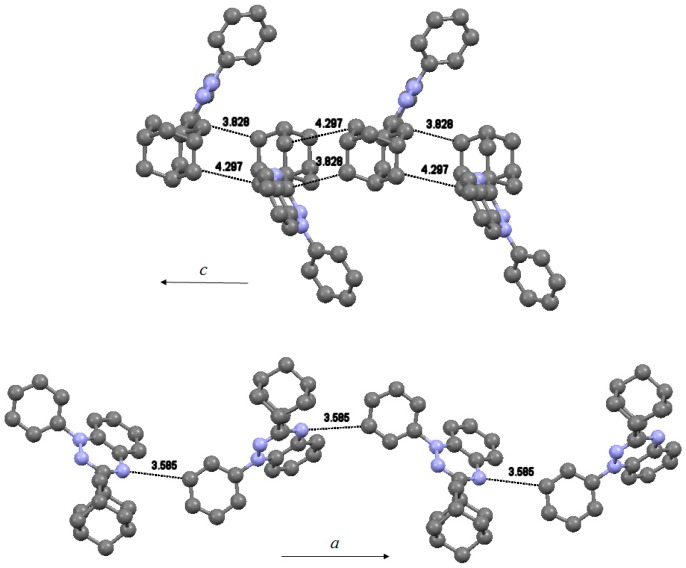
Solid-state packing of radical **4** demonstrating the formation of chains along *c*-axis (**top**) and along *a*-axis (**bottom**).

Each molecule in the asymmetric unit forms a linear chain of radicals at regular distances. Radicals in the chain running parallel to *c*-axis (Figure 4, top) have two close contacts, one between neighboring adamantyls [d_C46**…**C19_ = 4.297(5)°] and one between adamantyl and benzotriazinyl [d_C20**…**C1_ = 3.828(5)°]. Radicals within this chain are related by a 2-fold screw axis with direction (0, 0, 1) at 0,0,z and screw component (0, 0, ½). Radicals in the second chain running parallel to *a*-axis (Figure 4, bottom) are connected via a weak hydrogen contact between neighboring benzotriazinyls [d_N6**…**C34_ = 3.585(4)°]. This contact may propagate an antiferromagnetic interaction between the positive spin density on N6 and the negative spin density on C34. Carbon C34 has minimal orbital density in the SOMO, however, through spin polarization, it possesses significant negative spin density. The radical chains are related by two glide planes, one perpendicular to (0, 1, 0) with glide component (½, 0, 0) and one perpendicular to (1, 0, 0) with glide component (0, ½, ½). No formation of columns by *π*-stacked radicals was observed. The spin bearing benzotriazinyl moieties do not interact with each other. The related 3-(*tert*-butyl)-1-phenyl-1,4-dihydro-1,2,4-benzotriazin-4-yl was shown to crystallize in 1-D columns without π-π interactions between the benzotriazinyl moieties [5,20]. This radical demonstrated typical paramagnetic behavior as it followed the Curie-Weiss law with θ = −0.3 K [5].

### 2.3. Magnetic Susceptibility Studies

**Figure 5:**
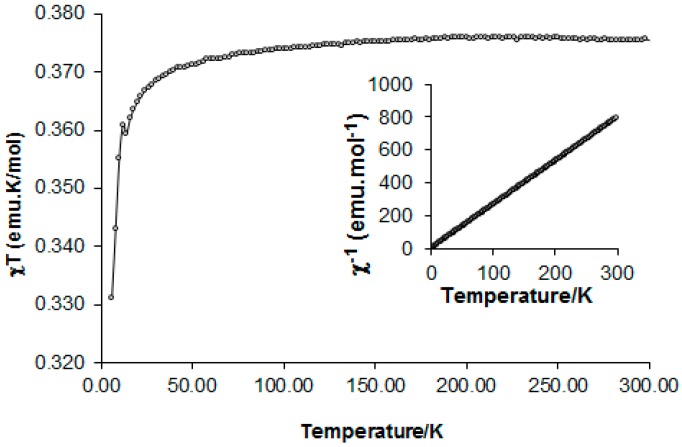
Temperature dependence of χT upon heating from 5 to 300 K. Inset: Curie-Weiss behavior, *C =* 0.377 emu K∙mol^−1^ and θ = −0.61 K.

Variable-temperature magnetic-susceptibility measurements on radical **4** were obtained using a SQUID magnetometer in the temperature region 5–300 K in an applied field of 0.4 T. Data were collected in both warming and cooling modes with no significant differences in sample susceptibility. The temperature dependence of χT is shown in Figure 5.

The inverse of molar susceptibility (1/χ) followed the Curie-Weiss law (Figure 5, inset) with *C* = 0.377 emu K∙mol^−1^ and θ = −0.61 K. On cooling from 300 K down to *ca*. 50 K, χT remained stable with a value of 0.375 emu K∙mol^−1^ expected for an *S* = ½ paramagnet. Below 50 K, χT gradually decreased as antiferromagnetic interactions along the a-axis chain (weak hydrogen d_N6**…**C34_ contacts) became dominant.

## 3. Experimental Sections

Synthetic procedure: The synthesis of radical **4** was published previously [10].

Instrumental analyses: Cyclic voltammetry (CV) measurements were performed on a Princeton Applied Research Potentiostat/Galvanostat 263A apparatus (Oak Ridge, TN, USA). The concentration of the benzotriazinyl radical **4** used was 1 mM in CH_2_Cl_2_. A 0.1 M CH_2_Cl_2_ solution of tetra-butylammonium tetrafluoroborate (*n*-Bu_4_BF_4_) was used as electrolyte. The electrolyte was dried for four days in the vacuum oven at 100 °C prior to the use. The reference electrode was Ag/AgCl and the scan rate was 50 mV/s. Ferrocene was used as an internal reference; the E_1/2_(ox) of ferrocene in this system was 0.352 V [26]. EPR spectra were recorded on a Bruker EMXplus X-band EPR spectrometer (Bruker, Billerica, MA, USA) at room temperature in dilute solution of CH_2_Cl_2_. For the EPR spectrum, the microwave power was in the region 5–70 mW with modulation frequencies of 50 or 100 kHz and modulation amplitudes of 0.5–1.0 G_pp_. Simulations of the solution spectrum was made using Winsim software [27]. X-ray data of **4** (CCDC 1474192) [28] were collected on an Oxford-Diffraction Supernova diffractometer, equipped with a CCD area detector utilizing Mo-Kα radiation (λ = 0.71073 Å). A suitable crystal was attached to glass fibers using paratone-N oil and transferred to a goniostat where they were cooled for data collection. Unit cell dimensions were determined and refined by using 4562 (3.85 ≤ θ ≤ 27.68°) reflections. Empirical absorption corrections (multi-scan based on symmetry-related measurements) were applied using CrysAlis RED software [29]. The structures were solved by direct method and refined on *F*^2^ using full-matrix least squares using SHELXL97 [30,31]. Software packages used: CrysAlis CCD [29] for data collection, CrysAlis RED [29] for cell refinement and data reduction, WINGX for geometric calculations [32] and Mercury 3.1 (CCDC, Cambridge, UK) [33]. The non-H atoms were treated anisotropically. The hydrogen atoms were placed in calculated, ideal positions and refined as riding on their respective carbon atoms. Magnetic properties were studied by using a Quantum Design SQUID MPMS_2_ field-shielded magnetometer (Quantum Design Inc., San Diego, CA, USA). The DC (direct current) magnetic moment was measured for 43 mg sample of radical **4**, placed in gelatin capsules held by polyethylene straw. The magnetic susceptibilities were measured in the temperature range of 5–300 K in an applied field of 0.4 T. Data were collected in both warming and cooling modes with no significant differences in sample susceptibility.

Crystal refinement data (**4**): C_23_H_24_N_3_, *M* = 342.45, Orthorhombic, space group *Pna21*, *a* = 18.5886(10) Å, *b* = 19.3115(9) Å, *c* = 9.9148(6) Å, α = 90°, β *=* 90°, γ = 90°, *V* = 35.59(2) Å^3^, *Z* = 8, T = 100(2) K, *ρ*_calcd_ = 1.278 g·cm^−3^, 2*θ*_max_ = 25. Refinement of 470 parameters on 5357 independent reflections out of 12978 measured reflections (*R*_int_ = 0.0438) led to *R*_1_ = 0.04761 [I > 2*σ*(I)], *wR*_2_ = 0.1154 (all data), and *S* = 1.050 with the largest difference peak and hole of 0.227 and −0.176 × 10^−3^, respectively.

## 4. Conclusions

Introduction of the bulky substituent adamantyl to the C-3 position of the 1,2,4-benzotriazinyl framework successfully inhibited the formation of *π*-stacked radical columns, but instead of promoting higher dimensional frameworks, only isolated radicals were obtained. This led to non-interacting *S* = ½ spins and a typical paramagnetic behavior. Our results indicate that there is a fine balance to be reached between steric perturbation and *π*-stacking interactions if materials with interesting magnetic and transport properties are to be developed.
